# Response of Retinal Blood Flow to Systemic Hyperoxia as Measured with Dual-Beam Bidirectional Doppler Fourier-Domain Optical Coherence Tomography

**DOI:** 10.1371/journal.pone.0045876

**Published:** 2012-09-18

**Authors:** René M. Werkmeister, Stefan Palkovits, Reinhard Told, Martin Gröschl, Rainer A. Leitgeb, Gerhard Garhöfer, Leopold Schmetterer

**Affiliations:** 1 Center for Medical Physics and Biomedical Engineering, Medical University of Vienna, Vienna, Austria; 2 Department of Clinical Pharmacology, Medical University of Vienna, Vienna, Austria; 3 Institute of Applied Physics, Vienna University of Technology, Vienna, Austria; Medical University Graz, Austria

## Abstract

**Purpose:**

There is a long-standing interest in the study of retinal blood flow in humans. In the recent years techniques have been established to measure retinal perfusion based on optical coherence tomography (OCT). In the present study we used a technique called dual-beam bidirectional Doppler Fourier-domain optical coherence tomography (FD-OCT) to characterize the effects of 100% oxygen breathing on retinal blood flow. These data were compared to data obtained with a laser Doppler velocimeter (LDV).

**Methods:**

10 healthy subjects were studied on 2 study days. On one study day the effect of 100% oxygen breathing on retinal blood velocities was studied using dual-beam bidirectional Doppler FD-OCT. On the second study day the effect of 100% oxygen breathing on retinal blood velocities was assessed by laser Doppler velocimetry (LDV). Retinal vessel diameters were measured on both study days using a commercially available Dynamic Vessel Analyzer. Retinal blood flow was calculated based on retinal vessel diameters and red blood cell velocity.

**Results:**

As expected, breathing of pure oxygen induced a pronounced reduction in retinal vessel diameters, retinal blood velocities and retinal blood flow on both study days (p<0.001). Blood velocity data correlated well between the two methods applied under both baseline as well as under hyperoxic conditions (r = 0.98 and r = 0.75, respectively). Data as obtained with OCT were, however, slightly higher.

**Conclusion:**

A good correlation was found between red blood cell velocity as measured with dual-beam bidirectional Doppler FD-OCT and red blood cell velocity assessed by the laser Doppler method. Dual-beam bidirectional Doppler FD-OCT is a promising approach for studying retinal blood velocities in vivo.

## Introduction

Ocular perfusion abnormalities are common and seen in a wide variety of diseases including glaucoma, diabetic retinopathy and age-related macular degeneration [1], [2], [3]. Nevertheless no gold standard method is available for quantifying blood flow in clinical use. Ultrasound color Doppler imaging is widely used for clinical studies, but information is limited to retrobulbar blood velocities and quantification of blood flow is hampered by the lack of diameter data [4]. Laser Doppler flowmetry and laser Speckle techniques can be used to assess optic nerve head and choroidal blood flow, but can provide only relative values of flow data [5], [6]. Retinal vessel diameters can be quantified using fundus cameras, but this technique provides no information on blood velocity [7]. Combined with fundus photography Laser Doppler Velocimetry (LDV) enables measurement of absolute blood flow in individual retinal vessels [8], [9], [10], [11]. By measuring all vessels entering the optic nerve head total retinal flow can be determined [10], [12], but long acquisition times and high patients' compliance are required.

Approximately 15 years ago first attempts were made to extract velocities by using optical coherence tomography (OCT) initially based on time domain OCT (TD-OCT) [13], [14]. Later approaches included variation of the coherence length [15] before phase sensitive detection allowed for extraction of velocities based on Fourier domain OCT (FD-OCT) [16], [17]. Several approaches were introduced in order to overcome the problem of the unknown Doppler angle, i.e. the angle between the incident probe beam and the vessel under study. This angle is required for measurement of absolute velocities, e.g. illumination of the sample with multiple probe beams [18], [19], extraction of the Doppler angle from multiple B-scans [20], [21] or extraction of the Doppler angle from a 3D volume [22], [23]. In order to overcome the problem in determining the angle between incident light and the vessel orientation, we introduced a dual-beam bidirectional Doppler FD-OCT system allowing for extraction of absolute blood velocity independently of the Doppler angle [24]. In the present study we investigated the response of retinal blood flow to 100% oxygen breathing using this system and compared the data with those obtained from a bidirectional LDV system.

## Methods

### Ethics Statement

The study protocol entitled “The validity of retinal blood flow measurement during hyperoxia in humans using Fourier domain CDOCT” was approved by the Ethics Committee of the Medical University of Vienna.

A total of 10 healthy subjects aged between 19 and 35 years participated in this study. The nature of the study was explained to all subjects and they gave written consent to participate. An ophthalmic examination, including slit lamp biomicroscopy and indirect funduscopy, was performed. Inclusion criteria were normal ophthalmic findings and ametropia of less than 1 diopter. In addition, a physical examination was done including electro cardiogram (ECG) and measurement of blood pressure (Infinity Delta, Dräger Medical Austria GmbH, Vienna, Austria) and pulse rate. Subjects were excluded if any abnormality was evident.

Two study days were scheduled for each subject. Both study days followed an identical schedule, but on one study day retinal blood velocities were measured using bidirectional LDV and on the other study day using dual-beam bidirectional Doppler FD-OCT. The sequence of the study days was randomized. For measurements in healthy volunteers pupils were dilated with one drop of tropicamide ( ''Mydriaticum Agepha®-eyedrops, 5 mg/ml tropicamide, AGEPHA GmbH, Vienna, Austria). During the study days a resting period of at least 20 minutes in a sitting position was scheduled to allow for stabilization of blood pressure and pulse rate. Thereafter, baseline measurement of retinal vessel diameter of a major temporal vein was done using the Dynamic Vessel Analyzer (DVA). This was followed by baseline measurements of retinal blood velocities at the same position where the diameter assessment took place with either LDV or dual-beam bidirectional Doppler FD-OCT, respectively, on both study days. Then a period of 100% oxygen breathing was scheduled and 15 minutes after the start of oxygen breathing retinal vessel diameters and retinal blood velocities were measured again. This time regimen was used because we have previously shown that the vasoconstrictor response to hyperoxia stabilizes after approximately 6 minutes [25]. On the second study day, the subjects crossed over to the other measuring device for retinal blood velocity. Dynamic vessel analyzer was also used on the second study day for the evaluation of vessel diameter. Oxygen (100%) (gases for human use, Messer; Vienna, Austria) was delivered through a partially expanded reservoir bag at atmospheric pressure. For gas delivery a face mask which covered mouth and nose connected to a two-valve system, preventing the subject from rebreathing was used. Intraocular pressure was measured after completion of blood flow assessment using applanation tonometry, blood pressure was measured non-invasively on the upper arm, and pulse rate was measured using a pulse oximeter (Infinity Delta, Dräger Medical Austria GmbH, Vienna, Austria).

### Dual-beam bidirectional Doppler FD-OCT

The setup of the dual beam bidirectional Doppler FD-OCT was described previously [24]. The basic principle of the instrument is to illuminate the vessel with two probe beams characterized by the wave vectors 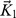 and 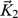. The beams are separated by their polarization properties and impinge onto the sample given by the velocity vector 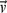 from two distinct directions. With this illumination and detection scheme, the measurement becomes independent of the exact angle of incidence of the probe beams. The phase shifts 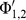, caused by the scattering of the impinging light at the red blood cells are given by 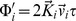 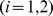 for the two beams, respectively. In this equation 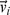 are the velocity vectors of the moving red blood cells (RBC) pointing in the direction of the corresponding probe beams 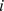. 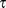 is the time span between two subsequent recordings, equaling the exposure time of the camera. The light backscattered from each probing channel is collected by two identical spectrometers, where the spectral modulations are detected as a function of frequency and the depth profile is calculated from an inverse Fast Fourier Transform (FFT)^−1^.

**Figure 1:**
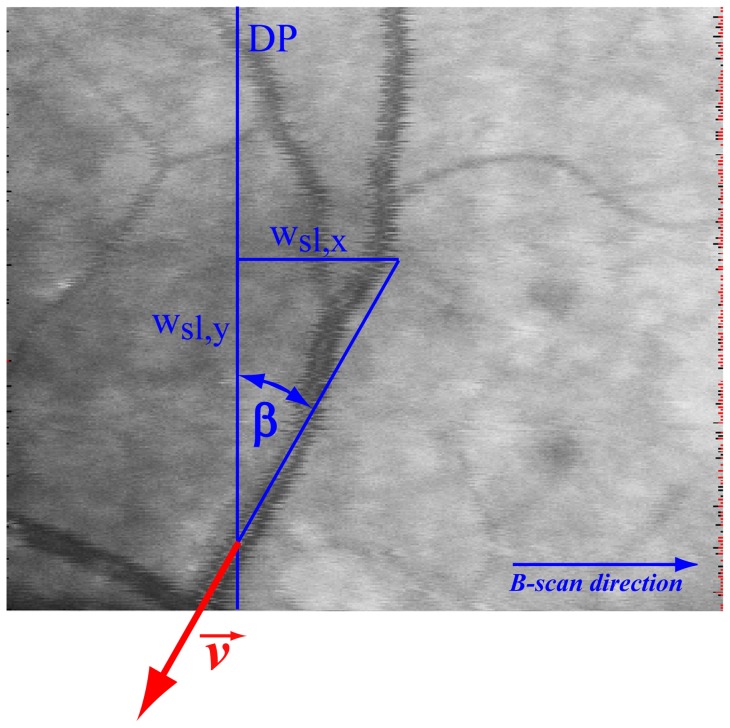
SLO fundus image of a venous junction. Angle 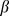 is determined from the slope of the tangent to the vessel at the measured location. w_sl,x_, w_sl,y_; image width in x- and y-direction. DP; detection plane.

Using simple trigonometry it can be shown that
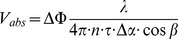
(1)where 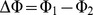. 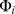 are the post-processed phase shifts, 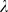 is the central wavelength of the light source, 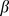 is the angle of the velocity vector with respect to the detection plane, 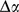 is the separation angle of the two beams at the retina and n is the group refractive index of blood, which was estimated as 1.37 [26]. With a parallel displacement of the two probe beams of 2 mm, the separation angle 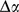 at the ocular fundus for a standard eye length of 24.2 mm [27] is 4.7 degree. For determination of the angle 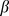 an additional scanning laser ophthalmoscope (SLO) detection arm measuring the back-reflected light is added to obtain an *en face* fundus image. This is achieved by placing the tangent to the vessel at the measured position and calculating the slope with simultaneous consideration of the true dimensions of the SLO image, i.e. 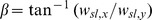 (Figure 1). Furthermore, the system comprises a custom built fundus camera that is coupled into the OCT sample arm by means of a dichroic mirror and allows for exact focal overlap of the two probe beams and inspection of the ocular fundus during the whole measurement time.

The system operates at a center wavelength 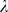 of 839 nm with a bandwidth 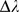 of approximately 52 nm, resulting in an axial resolution in air of 6 µm. The time period between two subsequent CCD recordings (A-scans) is 56 µs, resulting in a frame rate of 9s^−1^ with a lateral tomogram dimension of 2000 A-lines. The maximum velocity that can be measured in the regions of the optic nerve head without any wrapping artifacts is approximately 20 mm/s. When phase wrapping in the center of the vessels is corrected as outlined in detail below, the maximum detectable velocity can almost be doubled. The oversampling factors (OF) of the phase tomograms – defined as 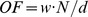 [28], where 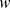 is the spot size, 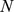 is the number of sampling points and 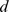 the geometric width of the tomograms–is 21 in our case. The power of both probe beams incident on the cornea was measured with 650 µW, which is below the ANSI (American National Standard Institute) limits for small source ocular exposure to a laser beam within the measuring time [29].

Even though the acquisition of a single tomogram in vivo takes only about a tenth of a second, any movement of the eye relative to the laser beam is a problem in phase-sensitive Doppler OCT, because it induces a phase-shift unrelated to blood flow. Therefore, the phase tomograms need to be corrected for those sample movements. For this purpose, the histogram-based approaches presented by Makita et al. [30] and Schmoll et al. [31] were optimized for movement correction in presence of large vessels. The modified algorithm uses the histogram not only for detection of the phase offset due to background motion, but also to distinguish between A-scans containing tissue only and A-scans containing both tissue and vessels.

After bulk motion correction the vessel position in the phase image is detected by convolution of the image with an elliptic template. The size of the template is set according to the dimensions of the selected vessel in the tomogram. The phase shift 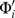 between adjacent A-scans is calculated as 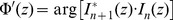, where 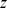 is the axial position, 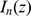 is the complex signal calculated by the inverse FFT of the *n* ^th^ scan, and 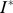 is the complex conjugate. Thus, the values for 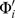 are in the range 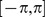. However, especially in arteries and large veins the velocity values can exceed the unambiguous velocity range leading to phase wrapping artifacts, i.e. a reversal of the measured flow direction in the center of the vessel. Such phase wraps can easily be corrected by determining the flow direction based on the phase shift values close to the vessel walls. As such, the phases in the center of the vessel are mapped into the range 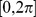 by 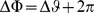 (if 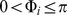) or into the range 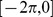 by 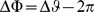 (if 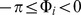). Double phase wraps did not occur in any of the measured veins in the present study. Another problem relates to fringe washout occurring particularly at high flow velocities. The missing Doppler data are reconstructed by applying a parabolic fit to the available data points [22]. It has been previously shown that the blood velocity profile differs from parabolic in retinal vessels [32]. However, since only missing data points are reconstructed while the original data stay untouched, the error introduced by this assumption is expected to be small.

**Figure 2:**
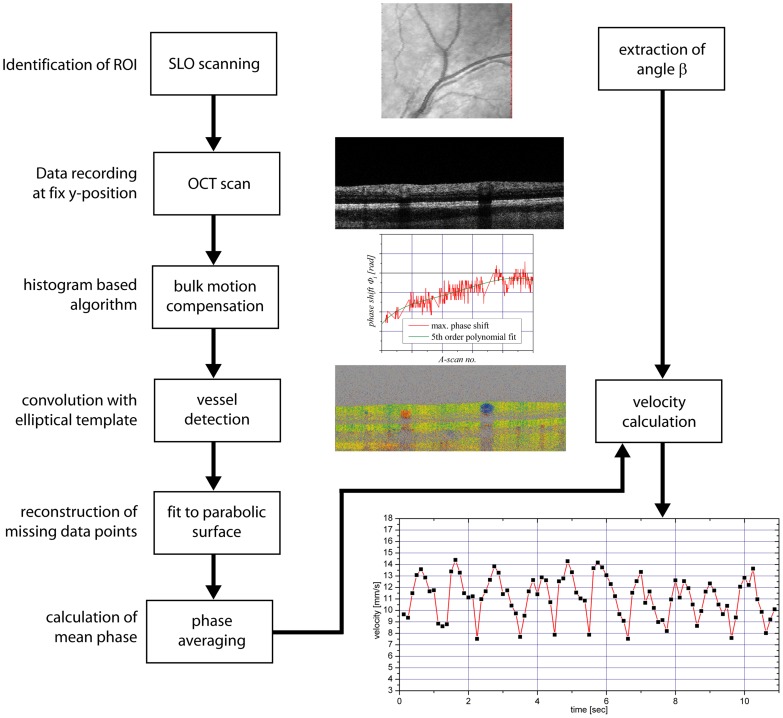
Flow chart for bidirectional Doppler FD-OCT. Flow chart explaining velocity extraction based on bidirectional Doppler FD-OCT.

For calculation of the average phase within the vessel cross section area, the approach introduced by Szkulmowska et al. was used [33]. Briefly, the method takes into account that the phase shifts 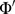 are randomly distributed around the actual value. Hence, when phase averaging is performed in the angular domain, the mean phase difference is underestimated particularly at high velocities. Therefore, the phase values are transformed to complex representation and the averaging is performed by calculating the argument of the complex sum. The average phase shift 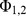 within a certain vessel is calculated for each frame of a series of 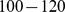 tomograms and for each detection channel separately. Afterwards, the actual phase difference 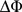 between the two probing channels is calculated by the simple subtraction 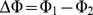. A diagram explaining the steps involved in signal analysis in shown in Figure 2.

### Laser Doppler Velocimetry and Dynamic Vessel Analyzer

For comparison, measurements were also done with a commercially available bidirectional laser Doppler velocimetry (LDV) system (LDV-5000, Oculix Inc., Arbaz, Switzerland) [9]. The principle of LDV is based on the optical Doppler effect. Laser light of a single-mode laser diode with a wavelength of 670 nm is scattered and reflected by moving erythrocytes leading to a broadened and shifted frequency spectrum. The frequency shift is proportional to the blood flow velocity in the retinal vessel. The maximum Doppler shift corresponds to the centerline erythrocyte velocity. The Doppler shift power spectra are recorded simultaneously for two directions of the scattered light. The scattered light is detected in the image plane of the fundus camera. This scattering plane can be rotated and adjusted in alignment with the direction of the velocity vector, which enables absolute velocity measurements. Blood flow values as well as velocity values were compared among LDV and Doppler OCT measurements.

In all these experiments retinal vessel diameters were measured in mydriasis with the Dynamic Vessel Analyzer (DVA, IMEDOS GmbH, Jena, Germany). This system comprises a fundus camera (FF 450, Carl Zeiss Meditec AG, Jena, Germany), a high-resolution digital video camera and a personal computer with analyzing software. For the determination of retinal vessel diameters, recorded images are digitized and analyzed in real-time with a frequency of 50 Hz. The system provides excellent reproducibility and sensitivity [7], [34]. After selection of the measurement location, the DVA is able to follow the vessels during movements within the measurement window.

### Effect of eye movements

**Figure 3:**
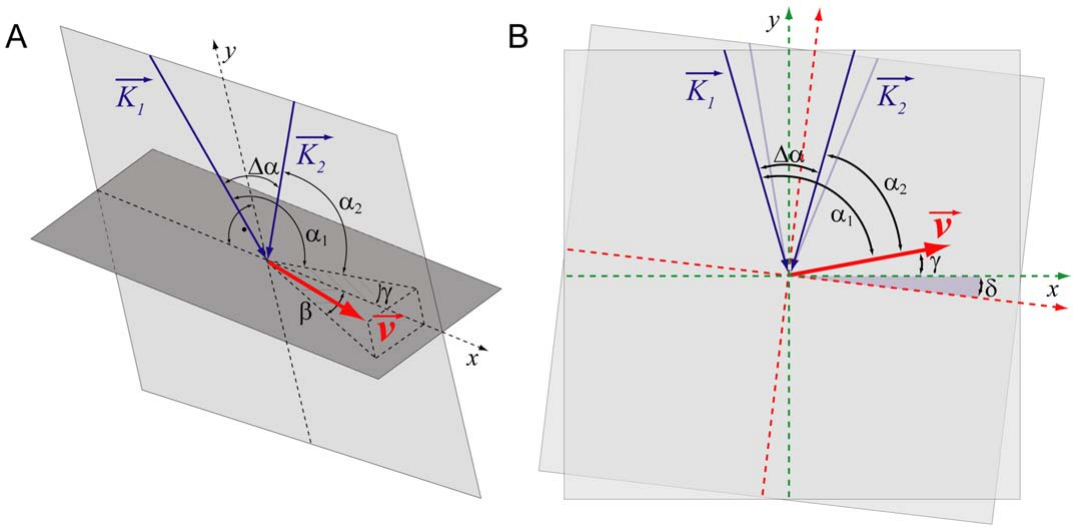
Geometrical situation at the posterior pole of the eye. 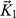
 and 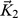, probe beam wave vectors; 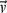, velocity vector; 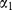 and 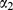, Doppler angles; 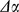, separation angle between probe beams; 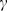, angle between 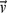 and the plane perpendicular to the detection plane; 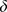, change in illumination angle due to eye movement.



The geometrical situation at the posterior pole of the eye is depicted in Figure 3(a). Here 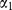 and 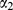 are two angles of the beams impinging on the retinal blood vessels, i.e. the Doppler angles, and 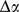 is the difference between these angles as mentioned above.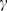 is the angle between the plane perpendicular to the optical axis of the illuminating beam and the velocity vector 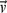, and 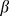 is the angle between the plane spanned by 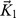 and 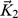 and the velocity vector 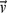. The phase shifts in the two channels can then be expressed as

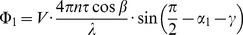
(2)

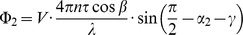



Assuming an emmetropic eye in which 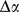 is 4.7 degrees or 0.082 rad, defining 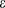 as 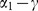 and considering that *in vivo* 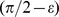 is very small, Equation 2 can be re-written as
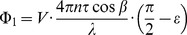
(3)

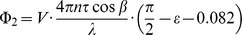
by replacing 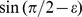 by the angle itself. As mentioned above, the difference in the phase is used to calculate the absolute velocity within the vessel (equation 1). Once absolute velocity is calculated, the angle 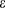 can be extracted based on equation 3. Since this can be done independently for both channels, the results of these calculations can serve as a proof for the validity of the dual beam bidirectional concept in the *in vivo* situation.

Assuming that the eye slightly moves during measurement and that accordingly 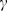 is changed into 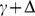 (red coordinate system in Figure 3(b)) and that the velocity changed from a value 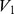 into a value 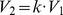. The phase shifts in the two channels then change to
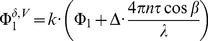
(4)

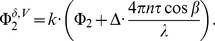



As such, while measuring the phase from each of these channels, it is unclear whether an observed change in phase is due to a change in blood velocity or a change in the angle of incidence. In the case that 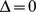 under optimal conditions, 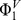 becomes 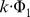 and 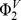 becomes 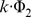. In this case the change in velocity as expressed by a factor 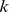, which is adequately mirrored in the data from both channels. If only 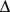 changes and 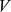 stays constant equation 4 simplifies to
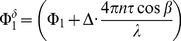
(5)

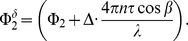



Note that in this case a change in velocity would be assumed relying on information from one channel only. Even if 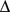 is very small; the effect on the phase might be huge depending on the absolute values of 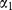 and 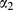. Assuming that 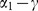 is 92.35 degrees and 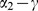 is 87.65 degrees a movement of only 1 degree (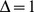 degree) would result in k-values of 1.43 and 0.57 for the data extracted from the two channels, respectively. The situation is even more severe in case the geometry is less symmetrical to the optical axis of the eye. If we assume that 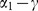 is 94.12 degrees and 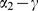 is 89.88 degrees a movement of 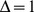 degree would result in k-values of 2.46 and −0.75 for the data extracted from the 2 channels, respectively. This means that data from channel one would erroneously imply that velocity has more than doubled, whereas data from channel 2 would even imply that velocity changed its direction. This is the case although absolute blood velocity stayed constant and 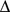 changed by 1 degree only.

Changes in the angle 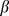 are much less critical. Assuming that 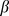 is 45 degrees, a change of 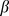 of 5 degrees only results in a k of 0.92 in both channels.

The same principle applies to bidirectional laser Doppler velocimetry, although the geometry is slightly different, because the eye is illuminated by one laser beam only, and the scattered laser light is detected in two distinct directions. Generally the frequency shift in the two channels is given by 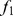 and 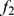, respectively, and absolute velocity can be calculated as 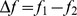.
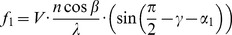
(6)

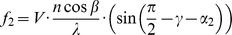



As such, small eye movements (changes in 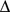) during LDV measurements have essentially the same impact as in bidirectional Doppler OCT, if one observes the signal in one of the two probing channels only.

### Data analysis

Velocity data as obtained with OCT technology are termed 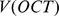 and data obtained with LDV technology are termed 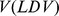. Generally, velocity values 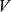 as presented in this article represent average values 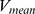 over the cross section of the vessel. In OCT, these data can be calculated from the actual measurements. In LDV, only the maximum Doppler shift can be extracted and 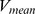 was calculated as 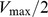 assuming a parabolic velocity profile [12]. Data at baseline are termed 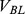 and data during oxygen breathing are termed 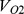. The same nomenclature was used for diameter measurements resulting in values 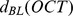, 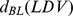, 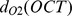 and 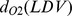. All diameter measurements were done with the DVA. OCT and LDV in this case simply denotes that measurements were done on the OCT and LDV study days, respectively.

Oxygen reactivity was defined as 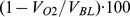. The same formula was used for diameter measurements. Total blood flow through the vein was calculated by 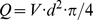. The angle 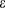 was extracted from equation 5 for the data obtained at both channels of the OCT system (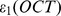 and 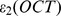). For this calculation small intervals of the phase shift time course were used, in which both phases showed only minor fluctuations. The same procedure was applied for the LDV system resulting in 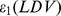 and 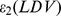.

Statistical comparisons were done using repeated measures ANOVA and paired t-tests. In addition, Bland Altman graphs were prepared for comparison of data. A two-sided p-value of less than 0.05 was considered the level of significance.

## Results

Table 1 summarizes the baseline data of the study population on the two study days. No difference was observed in blood pressure, pulse rate or vessel diameter. In addition, neither blood pressure nor pulse rate changed during 100% oxygen breathing (data not shown). Results of velocities, vessel diameters and blood flow during baseline and during 100% oxygen breathing are presented in Table 2 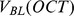 was slightly higher than 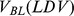 (p = 0.01). A tendency towards higher velocity values with OCT was also seen during hyperoxia but the difference between the 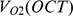 and 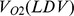 did not reach the level of significance (p = 0.06). There was no difference in vessel diameters on the two study days during both baseline and hyperoxia conditions (baseline 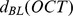 versus 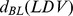 p = 0.86, hyperoxia 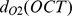 versus 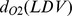 p = 0.55). As expected from these results, blood flow 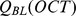 was also higher than 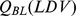 (p = 0.01). By contrast there was no significant difference between 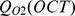 and 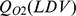 although a tendency towards higher values was observed when OCT was employed (p = 0.09). The oxygen reactivity in velocity was comparable between the two study days (OCT day: 32.6±17.2%, LDV day: 35.8±15.7%, p = 0.44). The oxygen reactivity in blood flow was also comparable (OCT day: 51.7±13.2%, LDV day: 51.1±11.9%, p = 0.90) between the two applied method.

**Table 1:** Baseline blood pressure, pulse rate and vessel diameters at the two study days.

	Day 1	Day 2	p-value
Systolic blood pressure (mmHg)	118±9	117±9	0.80
Diastolic blood pressure (mmHg)	67±6	66±6	0.71
Pulse rate (beats/min)	64±9	63±7	0.78
Retinal vessel diameter (µm)	141.0±18.8	140.1±19.1	0.86

**Table 2:** Velocity, diameter and blood flow data under baseline conditions and during 100% oxygen breathing measured on Doppler OCT or Laser Doppler Velocimetry study day.

	Baseline	O2	p-value
Velocity (OCT) [mm/s]	10.1±2.3	6.7±1.7	<0.001
Velocity (LDV) [mm/s]	9.2±2.3	5.9±1.9	<0.001
Diameter (OCT) [µm]	141±19	123±20	<0.001
Diameter (LDV) [µm]	140±19	122±17	<0.001
Blood flow (OCT) [µl/min]	10.3±2.9	5.0±2.2	<0.001
Blood flow (LDV) [µl/min]	9.0±2.7	4.2±1.8	<0.001

**Figure 4:**
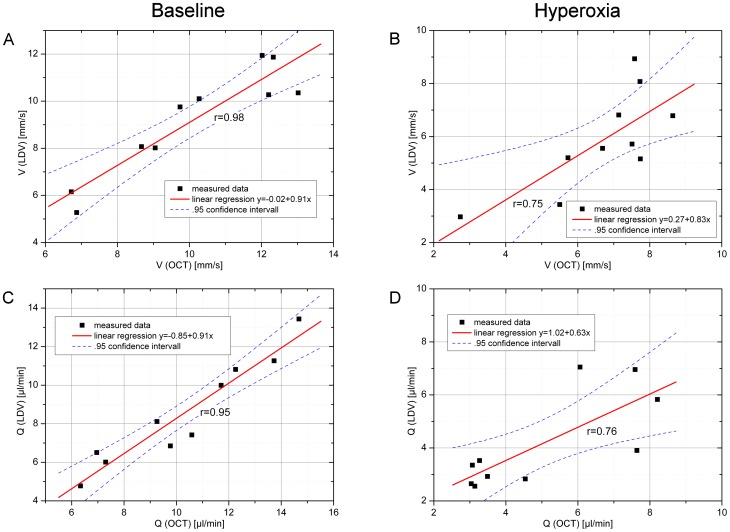
Correlation analysis for velocity measurements. Correlation between velocity and blood flow as assessed with LDV and OCT, respectively, during baseline conditions and during hyperoxia.

**Figure 5:**
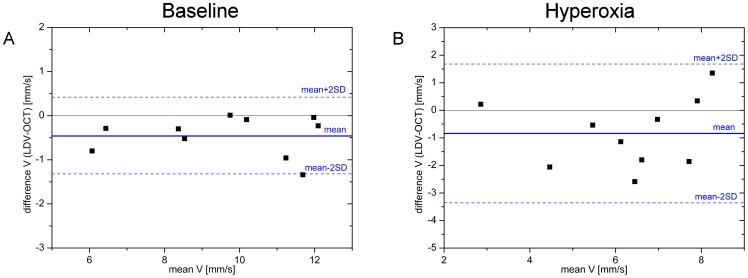
Bland-Altman of velocity measurements. Bland-Altman plot comparing velocity data as obtained with laser Doppler velocimetry and with bidirectional Doppler FD-OCT. Data are presented for baseline conditions as well as for conditions during systemic hyperoxia.

**Figure 6:**
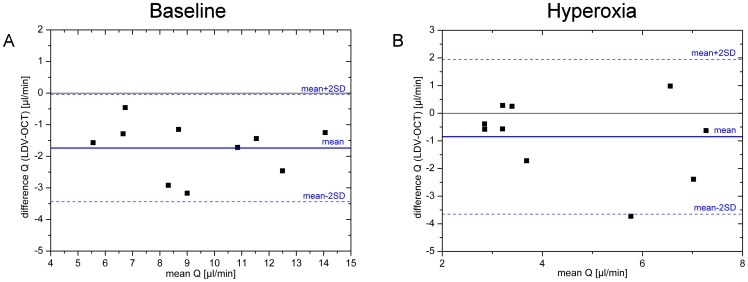
Bland-Altman plot of blood flow calculation. Bland-Altman plot comparing retinal blood flow data as calculated from measurements of blood flow velocity obtained either with laser Doppler velocimetry or with bidirectional Doppler FD-OCT combined with diameter measurements with the Dynamic Vessel Analyzer. Data are presented for baseline conditions as well as for conditions during systemic hyperoxia.

The correlation between 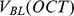 and 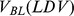 as well as 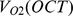 and 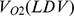 is presented in Figure 4. The degree of association was better during baseline conditions than during breathing 100% oxygen, although the correlation was highly significant under both conditions (p<0.01). The same applies to the correlation between 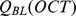 and 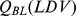 as well as 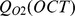 and 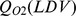 as shown in Figure 4. This is also reflected in the Bland Altman plots presented for 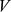 and 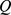 in Figures 5 and 6, respectively. Generally, all values were slightly lower when measured with LDV as compared to OCT. The difference never exceeded 3 mm/s for velocity and 4 µl/min for blood flow measurements.

**Figure 7:**
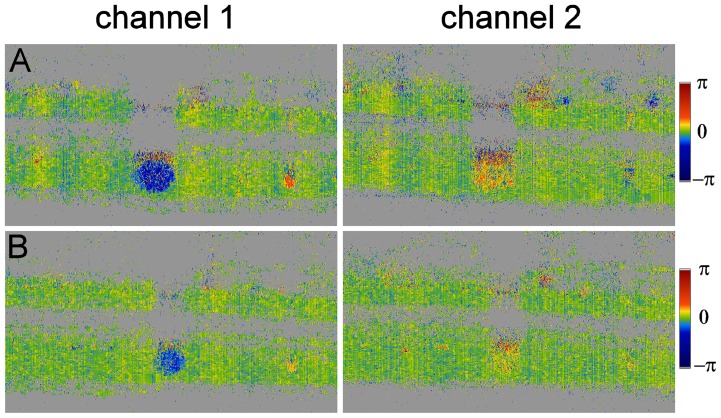
Phase tomograms at baseline and during hyperoxia. Sample OCT measurements as obtained in both channels during baseline conditions (A) and during breathing 100% oxygen (B). Vasoconstriction and reduction of velocity can be observed in the lower tomograms.

A sample measurement is presented in Figure 7. In both channels the reduction in velocity, but also the vasoconstriction in response to 100% oxygen breathing is clearly visible. The different colors indicate that in one channel the angle of incidence, i.e. the Doppler angle 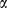, is below 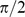, in the other channel above 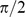. In channel 2 the angle is, however, obviously close to 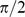.

**Figure 8:**
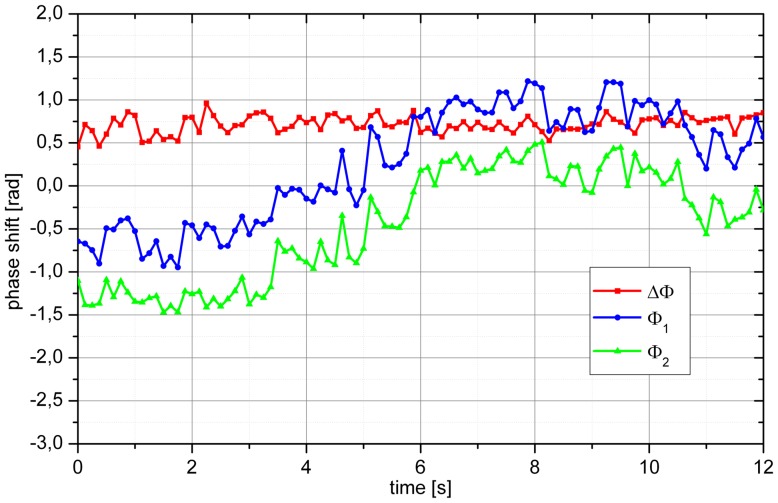
Sample phase extraction of a baseline measurement. Over the measurement period of 12 s the eye moved relative to the incoming laser beams resulting in pronounced changes in Ф_1_ and Ф_2_, but almost unchanged ΔФ.

**Figure 9:**
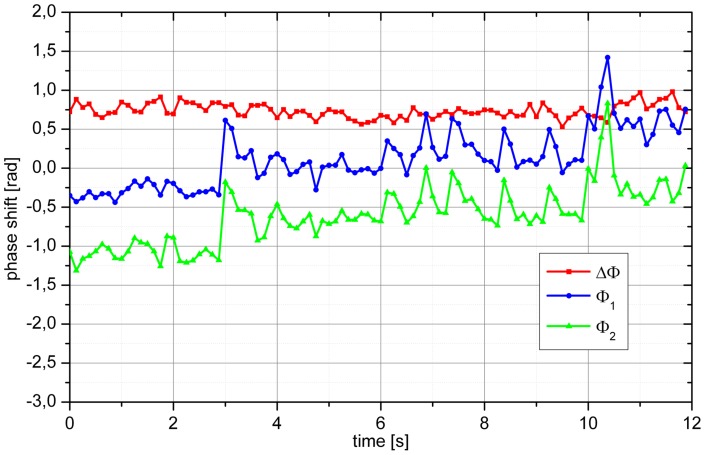
Sample phase extraction of a baseline measurement. Over the measurement period of 12 s several small eye movements can be seen resulting in pronounced changes in Ф_1_ and Ф_2_, but almost unchanged ΔФ. In addition, the eye also shows a slight overall movement resulting in shifts of Ф_1_ and Ф_2_ over time.

Figure 8 and Figure 9 present phase extractions from two baseline measurements. In the example shown in Figure 8 the phases 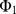 and 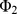 were almost stable for the first 2 seconds. Thereafter, 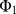 and 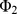 showed a continuous change, which was, however, parallel for both channels. This is also seen from the stability of 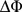 over time and as such represents a movement of the eye relative to the laser beams. Over time 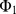 changed from −1.5 rad to 0.5 rad and 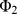 changed from −0.75 rad to 1.25 rad. In the example shown in Figure 9, we also observed a drift in 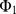 and 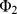. In addition, several spikes in 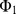 and 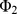 were detected. A total of 7 spikes were observed during the measurement period. During all these spikes, which may reflect saccade-like eye movements, 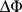 remained constant.

**Figure 10:**
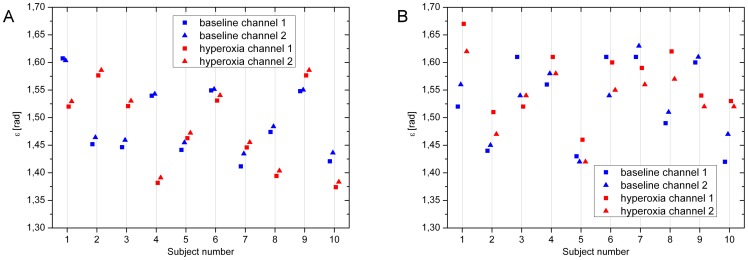
Calculation of angle 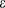. Angle 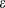 as calculated from measurements using OCT (red) and LDV (blue) from the two channels. Calculations were done at baseline conditions as well as during hyperoxia.

Figure 10 shows the calculation of 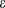 from 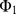 and 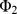 as presented in equation 3. Good agreement was found between the angles obtained at baseline in the OCT measurements (r = 0.999, p<0.001) and during 100% O_2_ breathing (r = 0.999, p<0.001). The correlation between the angles before and during O_2_ breathing was, however, weak and not significant (channel 1: r = 0.326, channel 2: r = 0.325). For LDV measurements, the correlation between the angles as calculated before (r = 0.855, p = 0.002) and during 100% O2 breathing (r = 0.937, p<0.001) was again significant, but the association was lower than in the OCT measurements. The correlations between the angles before and during O_2_ breathing were significant, but only for channel 2 (channel 1: r = 0.373, channel 2: r = 0.690, p = 0.027).

## Discussion

We present measurements of retinal blood flow velocity using bidirectional FD-OCT in healthy subjects during normoxia and hyperoxia and comparison with bidirectional LDV data. Generally, there is good agreement between the two techniques, but bidirectional FD-OCT offers several advantages that will be outlined in more detail below. Our data also indicate that single-beam techniques are extremely sensitive to movement artifacts. This makes it difficult to use them for measurement of absolute blood flow velocity, but also for quantifying changes in blood flow or blood flow velocity during stimulation.

Until the introduction of Doppler OCT, bidirectional LDV was the only technology capable of measuring absolute blood flow velocities in retinal vessels. Using this technique, absolute blood flow values in the retina have been published by several authors combing LDV with measurement of retinal vessel diameters. Riva and co-workers were the first to report measurements of total retinal blood flow with a value of 34±6 µl/min [10]. In subsequent publications, values between 37±5 µl/min and 80±12 µl/min were reported [11], [12], [35], [36], [37], [38], [39]. In the present study, good agreement was found between velocities as measured with LDV and OCT, although the values as obtained with the latter were slightly higher. Most likely, this is related to some methodological problems with the LDV system. Theoretically, the power spectrum from a cylindrical tube with a flowing fluid is a rectangle, in which the center line velocity corresponds to the maximum of the frequency shift [8]. In reality, however, the power spectrum is non-rectangular and the validity of center line velocity extraction depends on the algorithm to estimate the maximum of the frequency shift [9]. Related to this problem is the question on how to calculate the mean velocity based on measurements of maximum velocity. Riva and co-workers in their original work used a factor of 1.6 based on in vitro measurements [10]. In vivo measurements using a scanning LDV, however, indicated very little deviation from a parabolic profile [40]. As such, we have used a factor of 2 in subsequent publications [35], [41]. Recent work using an adaptive optics scanning laser ophthalmoscope, however, found much smaller values between 1.5 and 1.65 [32]. Using Doppler OCT, this assumption is less critical, because the actual velocity profile is measured. In our approach a theoretical assumption about the velocity profile is only required for reconstruction of missing data points.

Another problem with the Oculix LDV system is that during eye movements the laser beam may not hit the center of the vessel (unpublished observation). This may well lead to an underestimation of retinal blood flow velocities and most likely contributes to the lower velocity values as assessed with LDV. During states of vasoconstriction as induced by 100% oxygen breathing, this problem is obviously more severe. The Canon LDV system overcomes this problem by using an eye tracker system allowing for automatic motion correction [42], [43], [44].

The absolute blood velocities as obtained in the present study as well as the effect of 100% oxygen breathing are in good agreement with previously published data. A variety of previous studies reported on the effect of 100% oxygen breathing on retinal blood flow and provided comparable data as those obtained in the present study [25], [37], [45], [46], [47]. This vasoconstrictor response is required to prevent the retinal tissue from toxic hyperoxia [48]. When comparing the data as obtained on the two study days, one needs to consider that neither the baseline diameter nor the hyperoxia-induced vasoconstriction was similar on the two study days. As such, the deviations from perfect association seen in Figures 4–6 may arise from two distinct phenomena, namely imperfections in the measurement procedures as discussed above and physiological day-to-day variability.

The present data clearly indicate that bidirectional Doppler OCT measurements are insensitive to changes in the angle of incidence of the laser beam on the vessel. This is evident from our theoretical considerations but also from our measurements as presented in Figures 9 and 10. One might argue that for the quantification of relative changes in blood flow, as induced for instance during hyperoxia, no bidirectional system is required. Our data, however, indicate that this is not the case. As can be seen from our angle calculations presented in Figure 10, the angle of incidence changed in all subjects between baseline measurements and measurements during 100% oxygen breathing. One may assume that the problem of sensitivity to changes in the Doppler angle may get less severe with fast acquisition times as achieved for instance with swept source OCT [49]. This is in principle true, but it needs to be considered that blood velocity even in veins shows pulsatility over the cardiac cycle requiring continuous measurement over several heart cycles in order to get a stable value.

The good agreement between the values of the angle 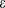 as calculated from the two channels of our bidirectional Doppler OCT system also indicates the validity of our model for the extraction of the absolute blood flow velocity. With LDV, the agreement in the calculations is less good. Here, one needs to consider that the bidirectionality of the system is achieved in the detection rather than the illumination pathway. These results most likely reflect the problems in extracting the maximum frequency of the LDV power spectrum as described above.

With the bidirectional LDV system as used in the present study, the maximum velocity that can be measured is determined by the readout rate of the cameras. At higher velocities, phase wrapping prevents adequate data extraction. This problem can be overcome by using faster cameras [22], [31], by using resonant Doppler OCT [50] or by using swept source OCT [49]. Other approaches were realized using Doppler OCT with the primary aim to visualize the retinal and choroidal microvasculature [30], [51], [52], [53].

The applications of bidirectional Doppler FD-OCT may be wide. In glaucoma there is a wide body of evidence that perfusion abnormalities are involved in the disease process [3], [54], [55]. A final proof of this concept is, however, lacking because up to now no technique for the measurement of retinal blood flow was available that could be employed in large-scale population-based studies. Another disease in which Doppler OCT may be of interest is diabetic retinopathy [56]. An early process in diabetic retinopathy is the partial loss of neurovascular coupling [57], [58], [59], [60], [61]. This may well contribute to neuronal loss in the diabetic retina, because of oxygen and glucose deprivation during neuronal activity. This concept has been elaborated in some details for the brain [62], but is relatively new for the retina [63], [64]. Preliminary data showing the effect of flicker light stimulation on retinal blood flow using Doppler OCT has been presented recently [65].

In conclusion, the data of the present study indicate that bidirectional Doppler FD-OCT is a valid technique for measuring retinal blood flow velocities. The technique is capable of measuring absolute blood flow velocities, but also to quantify the effect of changes in perfusion such as introduced by 100% oxygen breathing. The Doppler angle is very critical, because only small changes in this angle lead to pronounced changes in the extracted phase. Our in vivo measurements do, however, indicate that the bidirectional approach can be used to overcome this problem. The applications of this technique may be wide including studies in glaucoma and diabetic retinopathy.
